# Baseline Prolonged PR Interval and Outcome of Cardiac Resynchronization Therapy: A Systematic Review and Meta-analysis

**DOI:** 10.5935/abc.20180198

**Published:** 2018-11

**Authors:** Pattara Rattanawong, Narut Prasitlumkum, Tanawan Riangwiwat, Napatt Kanjanahattakij, Wasawat Vutthikraivit, Pakawat Chongsathidkiet, Ross J Simpson

**Affiliations:** 1 University of Hawaii Internal Medicine Residency Program, Honolulu, Havaí - EUA; 2 Department of Internal Medicine, Einstein Medical Center, Filadélfia - EUA; 3 Department of Internal Medicine, Texas Tech University, Texas - EUA; 4 Duke University Medical Center, Carolina do Norte - EUA; 5 The University of North Carolina, Chapel Hill, Carolina do Norte - EUA

**Keywords:** Heart Failure/complications, Heart Conduction System/physiopathology, Ventricular Dysfunction/complications, Cardiac Resynchronization/methods, Review, Meta-Analysis

## Abstract

**Background:**

Recent studies suggest that baseline prolonged PR interval is associated with worse outcome in cardiac resynchronization therapy (CRT). However, a systematic review and meta-analysis of the literature have not been made.

**Objective:**

To assess the association between baseline prolonged PR interval and adverse outcomes of CRT by a systematic review of the literature and a meta-analysis.

**Methods:**

We comprehensively searched the databases of MEDLINE and EMBASE from inception to March 2017. The included studies were published prospective or retrospective cohort studies that compared all-cause mortality, HF hospitalization, and composite outcome of CRT with baseline prolonged PR (> 200 msec) versus normal PR interval. Data from each study were combined using the random-effects, generic inverse variance method of DerSimonian and Laird to calculate the risk ratios and 95% confidence intervals.

**Results:**

Six studies from January 1991 to May 2017 were included in this meta-analysis. All-cause mortality rate is available in four studies involving 17,432 normal PR and 4,278 prolonged PR. Heart failure hospitalization is available in two studies involving 16,152 normal PR and 3,031 prolonged PR. Composite outcome is available in four studies involving 17,001 normal PR and 3,866 prolonged PR. Prolonged PR interval was associated with increased risk of all-cause mortality (pooled risk ratio = 1.34, 95 % confidence interval: 1.08-1.67, p < 0.01, I^2^= 57.0%), heart failure hospitalization (pooled risk ratio = 1.30, 95 % confidence interval: 1.16-1.45, p < 0.01, I^2^= 6.6%) and composite outcome (pooled risk ratio = 1.21, 95% confidence interval: 1.13-1.30, p < 0.01, I^2^= 0%).

**Conclusions:**

Our systematic review and meta-analysis support the hypothesis that baseline prolonged PR interval is a predictor of all-cause mortality, heart failure hospitalization, and composite outcome in CRT patients.

## Introduction

It has been widely accepted that surface electrocardiogram findings are associated with prognosis in congestive heart failure (HF) patients who have required cardiac resynchronization therapy (CRT), particularly the QRS complex. QRS duration and morphology is a well-established predictor of outcome among patients receiving CRT as well as selection criteria for CRT implantation according to the current guidelines of the American College of Cardiology/American Heart Association/Heart Rhythm Society.^1^

More recently, baseline PR interval has been invoked as an additional factor that may affect CRT outcomes.^2^ A prolonged PR interval is a marker of a ventricular substrate that is less amenable to resynchronization. It also reflects a combination of intrinsic intra-atrial and atrioventricular conduction which impacts diastolic filling time.^2^^,^^3^ There are no clear evidence and explanation why PR prolongation might contribute to the outcome of CRT patients. Nonetheless, there is controversial evidence in literature regarding the association between baseline PR prolongation and outcomes of HF patients who require CRT implantation. Some studies implied that PR prolongation was associated with higher morbidity and mortality amongst these patients,^2^^,^^4^^-^^7^ while others suggested it is associated with favorable outcomes.^8^^-^^10^ However, a systematic literature review and meta-analysis of the association between PR interval and CRT outcome have not been made.

We have first conducted a systematic literature review and meta-analysis to comprehensively analyze whether baseline PR prolongation in comparison with normal PR interval is associated with outcomes in CRT-dependent HF patients by assessing all-cause mortality, HF hospitalization rate, and composite outcome as our interest.

## Method

### Search strategy

Two investigators (NP and TR) independently searched for published studies indexed in MEDLINE and EMBASE databases from inception to January 2017 using a search strategy that included the terms “PR interval” and “cardiac resynchronization therapy” described in supplementary document 1. Only English language publications were included. A manual search for additional pertinent studies and review articles using references from retrieved articles was also made.

### Inclusion criteria

The eligibility criteria included the following:

1. Cohort study (prospective or retrospective) reporting incident of all-cause mortality, HF hospitalization, or composite outcome, after the CRT and the corresponding index date for controls.
2. Relative risk, hazard ratio, incidence ratio, or standardized incidence ratio with 95% confidence intervals or sufficient raw data for the calculation were provided.
3. Participants without PR prolongation were used as controls.

Study eligibility was independently determined by two investigators (NP and TR) and differences were resolved by mutual consensus. A Newcastle-Ottawa quality assessment scale was used to evaluate each study in three domains: recruitment and selection of the participants, similarity and comparability between the groups, and ascertainment of the outcome of interest among cohort studies.^11^

### Data extraction

A standardized data collection form was used to obtain the following information from each study: title of study, name of first author, year of study, year of publication, country of origin, number of participants, demographic data of participants, method used to identify cases and controls, method used to diagnose the outcomes of interest (all-cause mortality, HF hospitalization rate and composite outcome), and average duration of follow-up with confounders that were adjusted and adjusted effect estimates with 95% confidence interval and covariates that were adjusted in the multivariable analysis.

To ensure accuracy, all investigators independently performed this data extraction process. Any data discrepancy was resolved by referring back to the original articles.

### Statistical analysis

We performed a meta-analysis of the included cohort studies using a random-effects model. The extracted studies were excluded from the analysis if they did not present an outcome in each intervention group or did not have enough information required for continuous data comparison. We pooled the point estimates from each study using the generic inverse-variance method of Der Simonian and Laird.^12^ The heterogeneity of effect size estimates across these studies was quantified using the I^2^ statistic. The I^2^ statistic ranges in value from 0 to 100% (I^2^**<**25%, low heterogeneity; I^2^**=**25%-50%, moderate heterogeneity; and I^2^**>**50%, substantial heterogeneity).^13^ A sensitivity analysis was performed to assess the influence of the individual studies on the overall results by omitting one study at a time. Meta-regression was performed to explore source of heterogeneity. Publication bias was assessed using funnel plot and Egger’s regression test^14^ (p**<**0.05 was considered significant). All data analyses were performed using the Stata/SE 14.1 software from StataCorp LP.

## Results

### Description of the included studies

**Figure 1:**
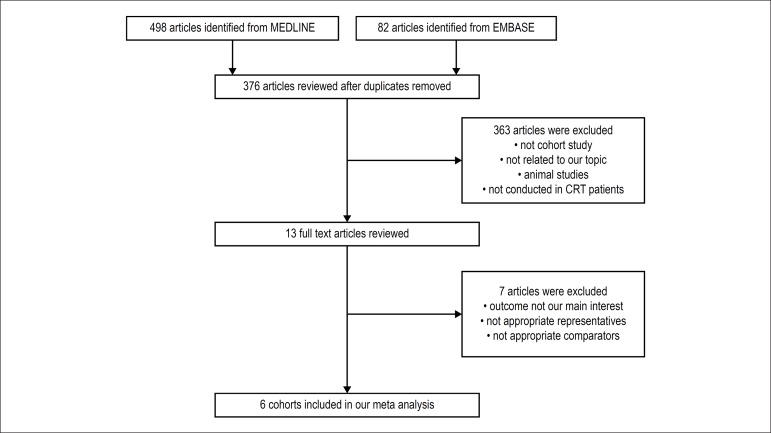
Search methodology and selection process.

Our search strategy yielded 580 potentially relevant articles (82 articles from EMBASE and 498 articles from MEDLINE). After exclusion of 204 duplicated articles, 376 underwent title and abstract review. Three hundred and seventy articles were excluded at this stage since they were not cohort studies, did not report the outcome of interest (incidence of death/HF hospitalization) or were not conducted in patients with CRT, leaving six for full-length article reviews. Therefore, six retrospective cohort studies with 17,432 normal PR and 4,278 prolonged PR patients were included in this meta-analysis. Figure 1 outlines the search and literature review process. The clinical characteristics and summary of the included studies are described in Table 1.

**Table 1:** The clinical characteristics and summary of the included studies

First author	Freidman	Januszkiewicz	Kronborg	Olshansky	Lee	Rickard
Country of Origin	USA	USA	Denmark	USA	USA	USA
Year	2016	2015	2010	2012	2014	2017
Study type	Retrospective cohort study	Retrospective cohort study	Retrospective cohort study	Retrospective cohort study	Retrospective cohort study	Retrospective cohort study
Participants description	Patients who underwent CRT (LVEF ≤ 35 and QRS ≥ 120)	Patients who underwent CRT (LVEF ≤ 35%, QRS > 120, NYHA III, IV)	Patients who underwent CRT	Patients who underwent CRT (LVEF ≤ 35, QRS ≥ 120 and NYHA III, IV)	Patients who underwent CRT (LVEF ≤ 35, QRS ≥ 120 and NYHA III, IV)	Patients who underwent CRT (LVEF ≤ 35, QRS ≥ 120)
Median duration of follow up (Months)	34	30.1	30	15.95	52.4	61.2
Definition of prolonged PR	≥ 230 ms	≥ 200 ms	≥ 200 ms	≥ 200 ms	≥ 200 ms	≥ 200 ms
Number of patients with prolonged PR	2906	125	208	638	204	197
Number of patients with not prolonged PR	15994	158	232	574	199	275
Mean age of patients	75.37	66.00	66.00	65.56	66.72	65.10
confounder adjustment	age, race, QRS, Intraventricular conduction, Non ischemic cardiomyopathy, NYHA, HF duration, eGFR, BUN, SBP	sex, RBBB, Ischemic cardiomyopathy, AF, medications	age, sex, HF aetiology, NYHA, DM, AF, ICD, LVEF	age, sex, NYHA, LVEF, LBBB, QRS, HR, SBP, DBP, ischemic status, comorbidities, medication	age, sex, ischemic cardiomyopathy, RV size, RV dysfunction, NYHA, MR grade, PASP, medication	age, sex, ischemic cardiomyopathy, LVEF, QRS, LBBB, Cr, NYHA

AF: atrial fibrillation; BUN: blood urea nitrogen; HF: heart failure; Cr: creatinine; CRT: cardiac resynchronization therapy; DM: diabetes mellitus; DBP: diastolic blood pressure; eGFR : estimated Glomerular infiltration; HR: heart rate; ICD: implanted cardiac defibrillator; LVEF: left ventricular ejection fraction; MR: mitral regurgitation; NYHA: New York Heart Association; PASP: pulmonary artery systolic pressure; RBBB: right bundle branch block; RV: right ventricular; SBP: systolic blood pressure.

### Quality assessment of the included studies

Newcastle-Ottawa scales of the included studies are described in Table 2. The Newcastle-Ottawa scale uses a star system (0 to 9) to evaluate the included studies on three domains: selection, comparability, and outcomes. Higher scores represent higher study quality. Intra-study risks of bias of the included studies are also described in Table 3.

**Table 2:** Newcastle–Ottawa scales of the included studies

Study	selection				comparability	outcome			
	representativeness	selection of the non-exposed cohort	ascertainment	end point not present at start	Comparability (confounding)	assesment of outcome	follow up duration	adequacy follow-up	total
Freidman	*	*	*	*	**	*	*	*	9
Januszkiewicz	*	*	*	*	**	*	*	*	9
Kronborg	*	*	*	*	**	*	*	*	9
Olshansky	*	*	*	*	**	*		*	8
Ying-Hsiang	*	*	*	*	**	*	*	*	9
Rickard	*	*	*	*	**	*	*	*	9

**Table 3:** Intra-study risks of bias of included studies

Study	Clear definition of study population?	Clear definition of outcomes and assessment?	Independent assessment of outcomes? (e.g. by third party)	Sufficient Follow-up duration?	Selective loss during Follow-up?	Limitations identified?
Freidman	Yes	Yes	Yes	Yes	No	Yes
Januszkiewicz	Yes	Yes	No	Yes	No	Yes
Kronborg	Yes	Yes	Yes	Yes	No	Yes
Kutyifa	Yes	Yes	Yes	Yes	No	No
Olshansky	No	Yes	Yes	No	No	Yes
Ying-Hsiang	Yes	Yes	No	Yes	No	Yes

### Meta-analysis results

**Figure 2:**
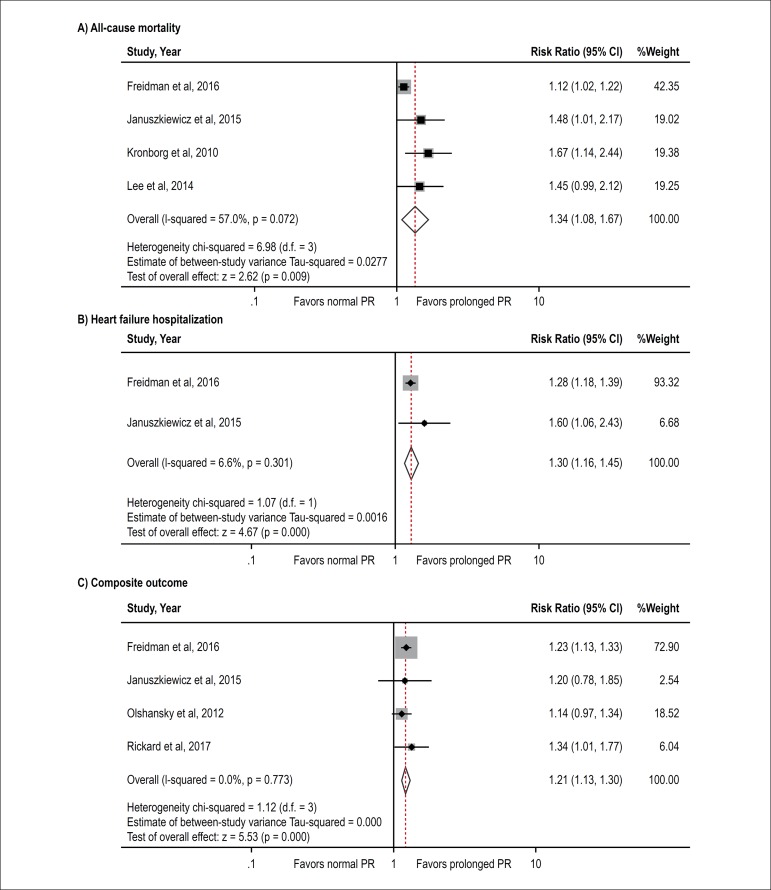
Forest plot of the included studies assessing the association between prolonged PR and risk of all-cause mortality (2A), HF hospitalization (2B), and composite outcome (2C).

Six studies^2^^,^^4^^,^^7^^,^^8^^,^^15^^,^^16^ from January 1991 to May 2017 were included in this meta-analysis. All-cause mortality rate is available in four studies^2^^,^^4^^,^^7^^,^^16^ that involved 17,432 normal PR and 4,278 prolonged PR. All four studies revealed an increased death rate among patients with prolonged PR interval but with of the four achieving statistical significance. The pooled analysis demonstrates a statistically significant increased risk of all-cause mortality in patients with prolonged PR interval compared to participants without prolonged PR interval with the pooled risk ratio of 1.34 (95 % confidence interval: 1.08-1.67, p < 0.01). The statistical heterogeneity was substantial with I^2^ of 57.0%. Forest plot of this meta-analysis is shown in Figure 2A.

HF hospitalization is available in two studies [2, 4] involving 16,152 normal PR and 3,031 prolonged PR. Both studies achieved statistical significance. HF hospitalization pooled risk ratio is 1.30 (95 % confidence interval: 1.16-1.45, p < 0.01). The statistical heterogeneity was low with I^2^ of 6.6%. Forest plot of this meta-analysis is shown in Figure 2B.

Composite outcome (all-cause mortality and HF hospitalization) is available in four studies^2^^,^^4^^,^^8^^,^^15^ involving 17,001 normal PR and 3,866 prolonged PR. All four studies revealed an increased death rate among patients with prolonged PR interval with two achieving statistical significance. In composite outcome, the pooled analysis also demonstrated a statistically significant increased composite outcome in CRT patients with prolonged PR interval compared to participants without prolonged PR interval with the pooled risk ratio of 1.21 (95% confidence interval: 1.13-1.30, p < 0.01). The statistical heterogeneity was low with I^2^ of 0%. Forest plot of this meta-analysis is shown in Figure 2C.

### Sensitivity analysis

To assess the stability of the results of the meta-analysis, we conducted a sensitivity analysis by excluding one study at a time. None of the results was significantly altered, indicating that our results were robust (supplementary document 2). However, after exclusion of Freidman et al.,^2^ the heterogeneity decreased from 57.0% to 0% (supplementary document 3).

Given moderate heterogeneity (I^2^ = 57.0%) among all-cause mortality meta-analysis results, meta-regression (supplementary document 3) showed non-significant changes in all-cause mortality in PR interval > 230 msec compared with PR interval > 200 msec with risk ratio of 0.73 (95% confidence interval: 0.43-1.23, p = 0.123).

### Publication bias

**Figure 3:**
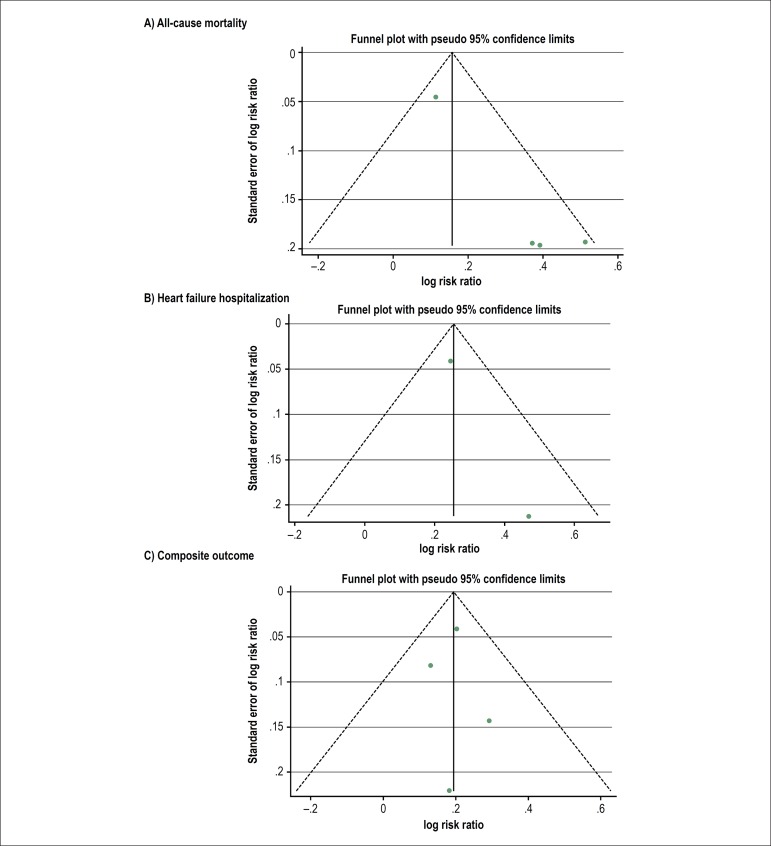
Funnel plot of prolonged PR and risk of all-cause mortality (3A), HF hospitalization (3B), and composite outcome (3C). Circles represent the observed published studies.

To investigate potential publication bias, we examined the funnel plot with pseudo 95% confidence limits of the included studies in assessing change in log risk ratio of death or composite outcome (Figure 3). The vertical axis represents study size (standard error) while the horizontal axis represents effect size (log risk ratio). From this plot, bias is present because there is asymmetrical distribution of studies on both sides of the mean. The Egger's test was significant (p < 0.05). However, using the trim and fill methods in the random-effects model, there was no difference of the imputed risk ratio and its 95% confidence interval.

## Discussion

The evidence provided in this systematic review and meta-analysis shows that a prolonged PR interval is significantly associated with an increased risk for all-cause mortality, composite outcome, and HF hospitalization of patients with CRT.

Prolongation of PR interval, also known as first-degree atrioventricular block, is independently associated with increased risk for mortality and atrial fibrillation in the general population.^17^ Even though correlation of PR interval with CRT response was conflicted in previous studies, our meta-analysis confirms the negative effect on clinical outcome in patients with prolonged PR interval. According to the Comparison of Medical Therapy, Pacing and Defibrillation in Heart Failure (COMPANION) trial, around 50% of patients with CRT have prolonged PR interval. In addition, patients with CRT and prolonged PR interval are more likely to have ischemic cardiomyopathy, wider QRS complexes, more severe right ventricular dysfunction, and renal diseases.^7^^,^^8^ The pathophysiology of PR prolongation causing adverse outcomes is explained by decreased ventricular filling time leading to decreased stroke volume. It can also induce ineffective mitral valve closure, causing diastolic mitral valve regurgitation, which is known to be associated with unfavorable outcomes in left ventricular dysfunction.^18^ The study results of Gervais et al.^6^ show that after CRT placement, there is a marked subsequent shortening of the mean PR interval, which suggests that CRT cures atrioventricular dyssynchrony.^6^ However, our result still shows worse outcome among patients with prolonged PR interval compared to normal PR interval. The reasons for PR interval affecting CRT outcome are uncertain. In general, prolonged PR interval reflects either intrinsic intra-atrial or atrioventricular conduction defect. Thus, CRT may facilitate AV synchrony to mitigate diastolic AV valve regurgitation and improve diastolic function.^19^ On the other hand, with the presence of intra-atrial conduction disturbance, CRT implantation could have deleterious impact on these patients as it shortens the appropriate PR interval and causes paradoxical effect, leading to worsening heart failure.^20^ Alternatively, PR prolongation may simply be a rough marker of “sicker” heart failure patients.^17^^,^^21^^,^^22^

In current heart failure guidelines, the duration of QRS, the type of bundle branch block and the presence of atrial fibrillation have been utilized as criteria for pacemaker device implantation.^23^ Also, CRT has a range of effects which has promoted interest in refining selection criteria for this important therapy. In our analysis, we imply hat the PR interval is a promising prognostic marker in patients with heart failure requiring CRT. Thus, PR interval may also be a valuable adjunctive selection criteria.

As our study has substantial heterogeneity in all cause mortality, we performed sensitivity analysis and found that after exclusion of Freidman et al.,^2^ the heterogeneity decreased from 57.0% to 0%. We concluded that the most likely explanation could be from the definition criteria of the recruited studies. Friedman is the only study that defined prolonged PR as more than 230 msec whereas every other study defined prolonged PR as more than 200 msec. Therefore, a meta-regression was conducted to investigate the statistical significance of PR definition affecting the results. However, meta-regression showed non-significant changes in all-cause mortality in PR interval > 230 msec compared with PR interval > 200 msec.

Our study has some limitations. Despite the fact that our funnel plot does not show biased data set, there are only six studies included in the analysis. In addition, PR prolongation is generally defined as PR interval exceeding 200 milliseconds. However, among the six included studies, there is only one study that defines prolonged PR interval as 230 ms and above.^2^ Given the total number of subjects, the heterogeneity of sample is small. While there are other possible predictor variables that are not included in this study, they were already analyzed in Rickard et al.^24^ Lastly, instead of using cardiac cause-specific mortality, all-cause mortality was used as outcome of interest in the included studies, which might overestimate the total outcome.

## Conclusion

In conclusion, among patients requiring CRT, prolonged PR interval is an independent indicator for all-cause mortality, HF hospitalization, and composite outcome. Our result suggests that PR interval should be considered as one of the important predictors of CRT response when addressing risk stratification.

## Supplementary Document 1 Search strategy and keywords

**Search strategy and keywords**

**EMBASE**

Searching term:

'pr interval' AND 'cardiac resynchronization therapy' AND [humans]/lim AND [english]/lim AND [clinical study]/lim

**Pubmed**

Searching term:

"pr interval"[All Fields] AND "cardiac resynchronization therapy "[All Fields] NOT "case report"[All Fields]
